# 

**Published:** 1826-10-01

**Authors:** 


### Contents

OF

No. 10. Vol. V. (NEW SERIES) for JULY, 1826.

[*No.* 26, *ANALYTICAL SERIES.*]

Art. I.

THE CIRCULATION AND ABSORPTION.

1. Dr. Barry's Researches on the Influence of Atmospheric Pressure on the Progression of the Blood in the Veins, and upon the Function of Absorption .. .. .. ..

2. Mr. Edward Hopley on the Syphhonic Theory .. .. } 313

II.

Mr. Fawdington on Melanosis.. .. .. .. .. .. .. .. 335

III.

Mr. Annesley's Topographical and Statistical Reports of the Diseases most prevalent in India.. .. .. .. .. .. 342

IV.

Dr. Mills on the Morbid Appearances exhibited in various Disorders of the Brain, on Dissection .. .. .. .. .. 350

V.

Mr. Travers on Dissection, or poisoned Wounds .. .. .. 363

(Second Article.)

VI.

MEDICO-CHIRURGICAL TRANSACTIONS OF EDINBURGH, VOL. II.

1. Mr. Wardrop on Exanthematous Ophthalmia .. .. .. 393

2. Mr. Hamilton on Beriberi .. .. .. .. .. .. .. 396

3. Dr. Berry's Description of Two Children united together .. 399

4. Mr. Alexander Watson on Chronic Inflammation of the Iris, 400

5. Dr. Ralph on the Yellow Fever of Barbadoes .. .. .. 401

6. Dr. Williams on Respiration and Animal Heat .. .. .. 401

7. Dr. Hamilton's Case of Extraction of Calculus by the Forceps, 402

8. Dr. Ramsay's Case of Extraction of Calculus by the Forceps, 403

9. Dr. Stevenson on the contagious Nature of Erysipelas.. .. 404

10. Mr. Howship on Mollities Ossium .. .. .. .. .. 405

VII.

Dr. George Gregory's Elements of the Theory and Practice of Physic (2d Ed.) .. .. .. .. .. .. .. .. .. 408

VIII.

M. Bretonneau on the Identity of Angina Maligna and Croup, with numerous Cases, Dissections, and a peculiar Mode of Treatment .. .. .. .. .. .. .. .. .. .. 419

### IX.

### PHRENOLOGY—PHYSIOGNOMY.

1. A System of Phrenology. By George Combe.. .. .. }
2. Phrenology, in connexion with the Study of Physiognomy. By G. Spurzheim, M. D. .. .. .. .. .. .. } 437

### X.

Novum Organum Medicorum; a new Medical Logic. By Professor Lanza—(concluded.) .. .. .. .. .. .. .. 470

### XI.

### Quarterly Periscope

OF

### PRACTICAL MEDICINE;

BEING

*THE SPIRIT OF THE MEDICAL JOURNALS,*

FOREIGN AND DOMESTIC.

Preface.. .. .. .. .. .. .. .. .. .. .. .. 486

1. Memoir on Gastralgic Affections mistaken for Gastrites .. 489

2. Dr. Monro on Spasmodic Affection of Muscular Tubes .. 500

3. M. Louis on Pericarditis, with Cases in illustration .. .. 504

4. Mr. Thompson's Case of distended Bladder .. .. .. 507

5. M. Guilbert on Leeching the Os Uteri, in certain Diseases .. 508

6. Mr. Hutchison on Imperforate Anus, with Commentaries .. 511

7. Dr. Lobstein on Kirronosis, an Affection of the Fœtus in Utero 517

8. Dr. Breschet and M. Geoffroy St. Hilaire on Extra-uterine Pregnancy .. .. .. .. .. .. .. .. .. .. .. .. 518

9. On the Extract of Garden Lettuce .. .. .. .. .. .. 522

10. On the Diagnosis of Pericarditis .. .. .. .. .. .. .. 523

11. On the Frequency of Loss of the Nose in Poland .. .. 523

12. Curious Instance of Vagitus Uterinus .. .. .. .. .. .. 524

13. Case of Periodical Aphonia .. .. .. .. .. .. .. .. 525

14. Case of extraordinary Pregnancy and artificial Anus .. .. 525

15. On the Hunger-cure, with Cases of its Application .. .. 527

16. Case of Worms voided from the Bladder .. .. .. .. .. 528

17. Neuralgia cured by Caustic .. .. .. .. .. .. .. .. 528

18. Conceptio Extra-uterina .. .. .. .. .. .. .. .. 529

19. Dr. Lesieur on Cutaneous Medication .. .. .. .. .. 531

20. Dr. Maclellan on Amputation of the Lower Jaw .. .. 532

21. A new Mode of curing Neuralgia, by Dr. Trevor .. .. 535

22. Successful Instance of the Lithontriptic Process .. .. .. 536

23. Expulsion of false Membrane, in Croup .. .. .. .. 538

24. Remarkable Instances of Epilepsy from Tænia .. .. .. 538

25. Mr. Allan on Specific Diseases and Specific Medicines .. 541

26. Instance of spontaneous Combustion .. .. .. .. .. 543

### XII.

### Periscope

OF

### AUTHENTICATED HOSPITAL REPORTS,

### *ENGLISH AND FOREIGN,*

WITH COMMENTARIES.

Introductory observations .. .. .. .. .. .. .. .. .. 545

1. M. Lisfranc's Memoir on White Swelling—*(La Pitié)* .. 546

2. Mr. Travers on Sloughing Ulceration—*(St. Thomas's Hospital)* 552

Mr. Green on Sloughing Ulceration *(Ditto.)* .. .. 553

Mr. Rose on Sloughing Ulceration—*(St. George's Hosp.)*.. 554

3. M. Velpeau on Tight Bandaging in Erysipelas, &c.—*(Hôp. Faculté.)* .. .. .. .. .. .. .. .. .. 554

4. Dr. Hewett on Follicular Ulceration—*(St. George's Hosp.)*.. 557

5. M. Lisfranc on Fractures of the Spine—*La Pitié.)* .. .. 560

6. Mr. Guthrie on Phlebitis after Amputation—*(Westm. Hos.)* 562

7. Professor Pfeuser on Œsophagitis, &c.—*(Bamberg Gen. Hos.)* 564

8. Dr. Chambers on Acute Rheumatism of the Joints—*(St. George's Hosp.)* .. .. .. .. .. .. .. .. .. .. 566

9. Dr. Bland's Hospital Reports—*(Hôp. Beaucaire)* .. .. 567

A. Scirrhus of the Pylorus *(Ditto.)* .. .. .. 567

B. On the Effects of Iodine *(Ditto.)* .. .. .. 568

C. On Carbuncle *(Ditto.)* .. .. .. 568

10. Mr. Shaw on Hernia—*(Middlesex Hospital)* .. .. .. 569

11. Observations on the Introduction of the Bougie .. .. .. 570

12. Lisfranc's Hospital Reports *(La Pitié.)* .. .. 570

A. Chloruret of Lime in Burns *(Ditto.)* .. .. 570

B. Excision of a Cancer in the Rectum *(Ditto.)* .. .. 571

C. On Morbid Sensibility of the Retina *(Ditto.)* .. .. 573

13. M. Geoffroy on Herpes Zoster—*(La Pitié)*.. .. .. .. 573

14. Mr. Bell on Aneurism—*(Middlesex)* .. .. .. .. .. 574

15. Dupuytren's Treatment of inverted Toe-nail—*(Hôtel Dieu.)* 575

16. Dr. Granville's Case of Gastrotomy—*(Westminster Infirmary)* 576

17. Dr. Latham's Case of Cerebral Affection—*(Bartholomew's)* 577

18. M. Velpeau on Humoral Pathology—*(Hôpital de Perfectionnement.)*.. .. .. .. .. .. .. .. .. .. .. 578

19. On the Treatment of Dysentery—*(Military Hospital, Chatham.)* .. .. .. .. .. .. .. .. .. .. .. 580

20. Paracentesis Thoracis—*(Philadelphia Infirmary.)* .. .. 582

21. Dr. Chambers on Masked Cerebral Affection—*(St. George's.)* 584

22. Ligature of the Thyroids for Bronchocele, Mr. Earle—*(Bartholomew's)* .. .. .. .. .. .. .. .. .. .. 585

23. Cancrum Oris Infantum, Dr. Coates—*(Philadelphia Asylum)* 586

24. Mr. Babington on Phagedenic Ulcers—*(Lock Hospital.)* .. 591

25. M. Roux's Surgical Reports *(Hôpital de la Faculté.)*.. 593

1. On Excision of the Tonsils *(Ditto.)*.. .. 594

2. Particular Treatment of Fistulæ *(Ditto.)*.. .. 594

3. Remarkable Case of deep-seated Abscess—*(Ditto.)* .. 595

26. Mr. Brodie on Disease of the Testicle—*(St. George's.)* .. 595
27. Remarkable Case of Polyphagia—*(Hôp. de Beaujon.)* .. 596
28. Mr. Chevalier's Case of Urinary Calculus—*(Westm. Disp.)*.. 597
29. M. Lisfranc's Treatment of Scirrhous Mammæ *(La Pitié.)*.. 598
30. Mr. Jeffreys on Injuries of the Head—*(St. George's)* .. 599
31. M. Velpeau's curious Cases of Paralysis—*(Hôp. de la Faculté.)* 602
32. Dr. Carmichael's Case of Disease of the Heart.. *(Middlesex.)* 605
Remarks on the above Case, with one in illustration .. .. 606

### XIII.

### ANALECTA.

1. Stethoscopic Indications of the Pregnant State, from the New Edition of Laennec, with Observations .. .. .. .. 607

2. Mr. Jowett on the Establishment of the Respiration in the New-born Child .. .. .. .. .. .. .. .. .. .. 610

3. Important toxicological Experiments, by Messrs. Edwards and Dumas .. .. .. .. .. .. .. .. .. .. .. 611

4. Curious Neuralgic Affection, by M. Hellis .. .. .. .. 612

5. Experiments on Pulmonary Exhalation, by Messrs. Breschet and Edwards .. .. .. .. .. .. .. .. .. 612

6. Cases of Transfusion, successful and unsuccessful .. .. 613

7. Case of fatal Placental Presentation .. .. .. .. .. 614

8. Mr. Earle's Case of Imperforate Anus, at Bartholomew's .. 615

9. A Case of fatal Dissection Wound .. .. .. .. .. .. 615

M. Gendrin's Experiments on Animals with Blood and other Fluids from diseased Subjects .. .. .. .. 616

10. Infanticide .. .. .. .. .. .. .. .. .. .. .. 617

11. Extra-uterine Conception .. .. .. .. .. .. .. 618

12. Rheumatism of the Heart cured by Acupuncture.. .. .. 619

13. Extirpation of the Ovarium.. .. .. .. .. .. .. .. 620

14. Acupuncture.. .. .. .. .. .. .. .. .. .. .. 621

15. Congenital Blindness cured at an advanced Age .. .. .. 622

16. Remarkable Disease of the Heart.. .. .. .. .. .. .. 623

17. Injuries of the Thorax .. .. .. .. .. .. .. .. .. 625

### XIV.

Bibliographical Record .. .. .. .. .. .. .. .. .. 626

### XV.

### INTELLIGENCE, ETC.

Report of Associated Apothecaries .. .. .. .. .. .. .. 630

On Uterine Hæmorrhagy .. .. .. .. .. .. .. .. .. .. 632

New Regulations of the College of Surgeons .. .. .. .. 633

Reply to a "Physician," on a Delicate Subject.. .. .. .. 635

Notice Respecting Dr. Paris's Work on Diet .. .. .. .. 636

### XVI.

### EXTRA-LIMITES.

Dr. C. Clarke's Observations on the Smyrna Fever .......... 637
Additional Subscribers ...................................... 644

